# A note on wildlife poisoning cases from Kerala, South India

**DOI:** 10.1007/s10344-018-1218-6

**Published:** 2018-09-22

**Authors:** Sreejith Radhakrishnan

**Affiliations:** 1 Periyar Tiger Reserve, Department of Forests and Wildlife, Thekkady, Kerala India; 2 Department of Infectious Disease Epidemiology, Imperial College London, St. Mary’s campus, Norfolk place, London, W2 1PG UK

**Keywords:** Wildlife, Poisoning, Kerala, Carbofuran, Rodenticide, Neonicotinoid, Endosulfan

## Abstract

Wildlife poisoning is an important conservation threat for endangered species in India. There are no publications in the scientific literature that identify the specific poisons or chemicals involved in wildlife poisoning cases from the state of Kerala. In this report, all cases of wildlife mortality recorded between 2011 and 2013 at the office of the Assistant Forest Veterinary Officer, Periyar Tiger Reserve in Kerala were reviewed and cases where poisoning was considered as a differential diagnosis were identified. Specific poisons or chemicals were identified in three cases, while in a fourth, poisoning was determined to have occurred based on physical traces of the poison in gut contents. The poisons identified include carbofuran (a carbamate pesticide) in a bonnet macaque (*Macaca radiata*), warfarin (a rodenticide) in a mortality event involving four wild boars (*Sus scrofa*), endosulfan (an organochlorine pesticide) toxicity in a gaur (*Bos gaurus*) and imidacloprid (a neonicotinoid pesticide) toxicity in a wild adult Asian elephant (*Elephas maximus*). This communication thus reports for the first time on the specific chemical compounds identified in wildlife poisoning cases from Kerala state and argues for greater regulation of the sale and use of such toxic compounds in India.

**Electronic supplementary material:**

The online version of this article (10.1007/s10344-018-1218-6) contains supplementary material, which is available to authorized users.

## Introduction

Poisoning is an important cause of wildlife mortality, and in some instances has been responsible for extensive population declines (Green et al. 2004). Primary exposure to poisons occurs when wildlife is intentionally poisoned for hunting (Ogada 2014) or due to human-wildlife conflict (Venkataramanan et al. 2008; Ragothaman and Chirukandoth 2012). Accidental poisoning may occur by secondary exposure to poisons in the environment or via contaminated food sources. Examples include pesticide residues in Irrawady dolphins living in contaminated water bodies (Kannan et al. 2005) and Sarus cranes consuming monocrotophos-treated grains (Pain et al. 2004), respectively. In all such instances, non-target wildlife may also be unintentionally poisoned when they scavenge on carcasses containing high concentrations of poisons (Kalaivanan et al. 2011; Molenaar et al. 2017).

Although the threats posed by intentional or accidental poisoning to wildlife in India have long been recognised (Spillett 1967), statistics on the proportion of wildlife deaths attributable to poisoning are lacking. Poisons or chemicals detected in wildlife in India include organochlorine (OC) (Kannan et al. 2005; Pathak 2011), organophosphate (OP) (Pain et al. 2004; Kalaivanan et al. 2011) or carbamate pesticides (Venkataramanan et al. 2008) and rodenticides (Ragothaman and Chirukandoth 2012). Ragothaman and Chirukandoth (Ragothaman and Chirukandoth 2012) listed 15 separate incidents of pesticide poisoning in wildlife between 2000 and 2010, including six instances (40%) of OP and four instances (27%) of carbamate pesticide poisoning.

However, wildlife mortality due to poisoning may be under-reported in India, either because poisoning is not considered as a possible cause; carcasses are found in advanced stages of decomposition or cases are lost to follow up after submission of tissue samples to testing laboratories (personal observation). This is true in the case of wildlife mortality reporting from the south Indian state of Kerala with its rich biodiversity owing to the Western Ghats, a global biodiversity hotspot which runs across the entire length of the state (Gunawardene et al. 2007). A search of the scientific literature on five databases (PubMed, Web of Science, Scopus, Ovid, ProQuest) and Google Scholar using the search terms “wildlife”, “poison*” and “Kerala” did not find any publications that identified the specific poison or chemical implicated in suspected poisoning events in the state. The four publications that mentioned wildlife poisoning in Kerala used generic terms such as ‘rodenticides’ (Jayahari and Jayson 2007), merely identified poisoning as the potential or confirmed cause of death (Cheeran 2007; Rohini et al. 2015) or provided anecdotal evidence (e.g. poisoning of elephants by ‘Folidol’, an OP pesticide) (Spillett 1967). Knowledge of the specific chemical compounds involved in wildlife mortality events is the first step in enabling government agencies to implement regulatory measures that can prevent incidents of poisoning or help populations that have suffered declines to recover (e.g. impact of the diclofenac ban on vulture recoveries) (Paudel et al. 2016). To fill this knowledge gap, this report provides details of the specific poisons/chemical compounds detected in wildlife mortality events from Kerala over a 2-year period.

## Methods

Periyar Tiger Reserve (PTR) in central Kerala has the services of a full-time wildlife veterinarian designated as Assistant Forest Veterinary Officer (AFVO). The AFVO deals with treatment of ill and injured wildlife, assists in human-wildlife conflict resolution and conducts necropsies and disease surveillance in PTR and adjoining wildlife reserves. For this report, all cases of wildlife mortality recorded between January 2011 and March 2013 at the AFVO’s office were reviewed. Cases were identified where poisoning was considered as a differential diagnosis based on circumstantial evidence, necropsy lesions or the need to rule out poisoning as a cause of death when no lesions were identifiable in decomposed carcasses. Records of mortality events prior to and after this period were not immediately accessible and have not been included in the review.

## Results and discussion

Eleven separate wildlife mortality events recorded between January 2011 and March 2013 met the selection criteria mentioned above. In ten instances, post-mortem tissue samples were submitted in saturated salt solution for toxicological analyses to the Regional Chemical Examiners Laboratory (RCEL), Ernakulam, one of three government laboratories in Kerala providing toxicological testing services free of charge to government departments (Chemical Examiner’s Laboratory Department 2017) (Table S1). Test results were reported as positive or negative for a specific poison after analyses of processed samples by thin layer chromatography (TLC) and a second method (gas chromatography–mass spectrometry or ultraviolet spectrophotometry). Detection of coloured spots with a retardation factor (*R*_f_) value equal to that of a reference compound during TLC confirmed presence of the poison. Specific poisons/chemicals were detected in three cases, although concentrations were not reported. In the other seven instances, negative test results were reported. In a fourth case where tissue samples were not submitted for analyses, poisoning was determined to be the cause of death based on lesions and physical presence of the chemical compound in gastric contents. These four cases are described in detail below.

1. Imidacloprid in an Asian elephant

**Fig. 1:**
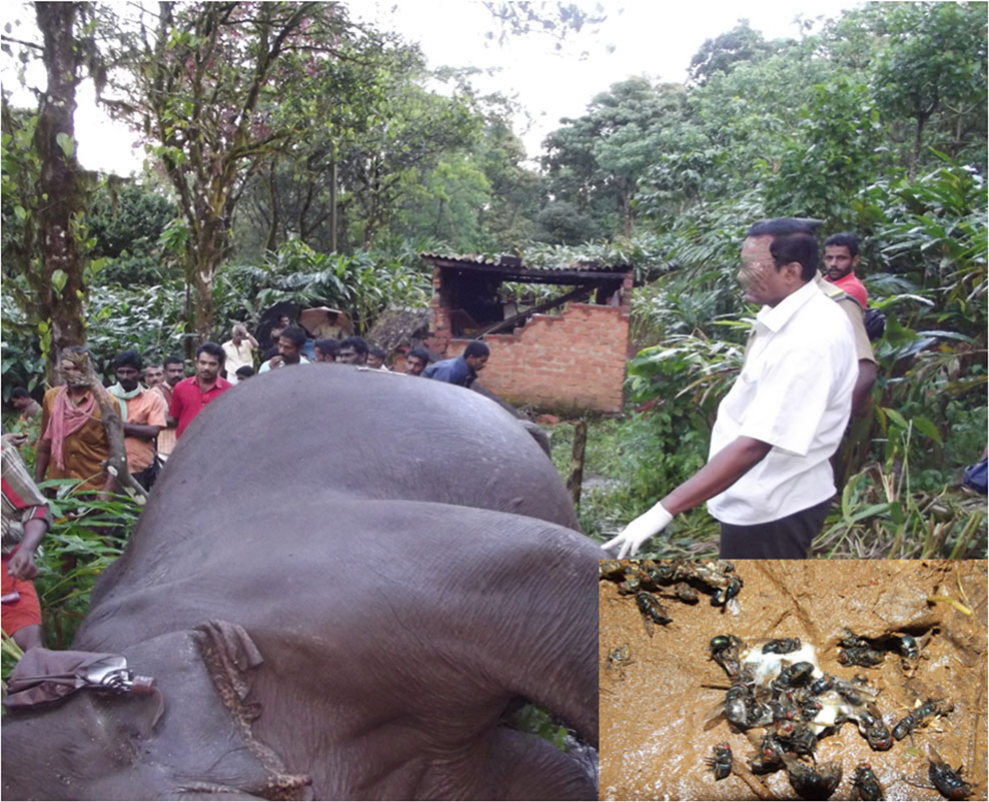
Elephant (*Elephas maximus*) carcass found within a cardamom plantation, next to a demolished shed used to store pesticides. Masses of dead flies were found near the elephant carcass and inside the oral cavity (inset)

A wild adult female Asian elephant (*Elephas maximus*) was found dead in a commercial cardamom plantation adjoining a forest reserve in Ayyappankovil range, Kattappana in November 2011. The carcass lay next to a demolished shed that had been used to store and prepare pesticides including imidacloprid (a systemic neonicotinoid pesticide), quinalphos and chlorpyriphos (OP pesticides). The elephant was suspected to have consumed water from a barrel which was used to dilute pesticides prior to spraying, whereby it may have been poisoned. Lesions observed included marked lingual and corneal cyanosis, diffuse pulmonary haemorrhage and hepatomegaly with subcapsular haemorrhages. The presence of large numbers of dead or moribund flies in the elephant’s mouth and around the carcass (Fig. 1), as well as dead gastric bots (commonly of the oestrid fly *Cobboldia elephantis)* in the stomach pointed to the likelihood of pesticide poisoning. Imidacloprid was detected in hepatic tissue and gut contents submitted to the RCEL.

Poisoning with neonicotinoid pesticides has been reported in humans (Cimino et al. 2017), domestic animals (Caloni et al. 2016) and small vertebrate wildlife such as birds and fish (Gibbons et al. 2015). Exposure to pesticides, including imidacloprid, was suspected to be associated with the occurrence of congenital deformities and reproductive problems in chimpanzees and baboons in Uganda (Krief et al. 2017). However, to the author’s knowledge, this is the first report of mortality of a large wild mammalian species linked to imidacloprid consumption. This highlights the need for further studies on the use of neonicotinoid pesticides and their impacts on wildlife and the wider environment. Such studies, sorely lacking in the Indian context, are urgently needed given the accumulating evidence globally of their role in population declines of bees as well as non-target invertebrate species (Pisa et al. 2015).

2. Endosulfan in a gaur

In August 2012, an adult male gaur (*Bos gaurus*) carcass was found in PTR in a forest patch on the border between Kerala and the adjoining state of Tamil Nadu, abutting a large commercial tea plantation. As the carcass was partially decomposed and lay close to the plantation where pesticide use was likely, samples of hepatic tissue and gut contents were submitted for toxicological analyses. Endosulfan, a toxic OC pesticide, was detected in all tissue samples. Given the state of decomposition of the carcass and absence of information on tissue concentrations of endosulfan, it was not possible to attribute death to pesticide exposure. It was considered more likely that long-term exposure occurred by consumption of pesticide-laden foliage or water around the plantation.

3. Carbofuran in a Bonnet Macaque

**Fig. 2:**
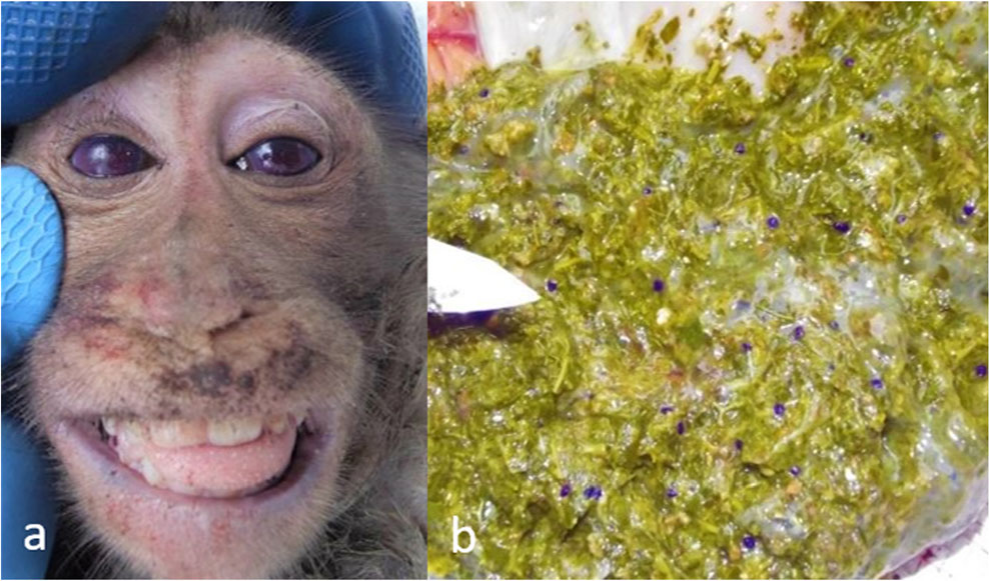
**a** Ocular cyanosis in a bonnet macaque (*Macaca radiata*). **b** Dark purplish-blue granules of carbofuran mixed with gastric contents

The carcass of an adult female bonnet macaque (*Macaca radiata*) was found on the terrace of a building in Kumily town, adjoining PTR, in October 2011. Necropsy lesions included severe ocular cyanosis and pulmonary congestion and marked splenomegaly. The stomach was filled with undigested food mixed with numerous dark purplish-blue granules (Fig. 2), tentatively identified as carbofuran, a carbamate pesticide widely available in Kerala and sold under various trade names (e.g. ‘Furadan’) (Cheeran 2007; Ragothaman and Chirukandoth 2012). Based on these findings, the macaque was suspected to have died of carbofuran poisoning after consuming pesticide-laced food. However, no tissue samples were submitted for toxicological analyses.

Substantial populations of bonnet macaques thrive in regions of Kerala adjoining wildlife habitat. They are generally viewed as pests and forest department officials often deal with complaints from the public when macaques steal food from local homes or restaurants, damage property or display aggression (Rohini et al. 2016). Incidents of deliberate poisoning of macaques are thus not uncommon (Ragothaman and Chirukandoth 2012).

4. Warfarin in wild boars

In September 2012, carcasses of four wild boars (*Sus scrofa*) of various ages were found in PTR. No distinct necropsy lesions were identifiable as all were actively decomposing. Poisoning was suspected since the carcasses were found over two days and near each other, close to office buildings and residential quarters. Analysis of hepatic tissue and gut contents detected warfarin, a first-generation anticoagulant rodenticide. It was unclear how the boars may have been exposed to warfarin. As they were known to frequent the area around the offices and quarters in search of food, malicious poisoning was considered likely, although accidental exposure (e.g. consumption from a carelessly discarded container while scavenging) could not be ruled out.

Wild boars frequently cause extensive agricultural losses in Kerala (Rohini et al. 2016) and retaliatory poisoning is commonly reported (Kalaivanan et al. 2011). They also scavenge food waste from garbage dumps close to human habitation, posing a potential risk to people. In 2011, the Government of Kerala decided to allow farmers who had incurred losses to apply for permits to kill wild boar, although this decision has been contested (Gopakumar et al. 2012).

In all the cases reported here, lack of information on concentrations of detected poisons makes it difficult to conclusively establish poisoning as the cause of death. Despite this, the findings are significant as they form the first report of specific poisons identified in wildlife mortality events in Kerala. In doing so, they add to the literature on wildlife poisoning in India and globally.

The report also reinforces the need for considering poisoning as a differential diagnosis in investigations of wildlife mortality events, irrespective of whether the primary cause of mortality can be established or not. This is particularly relevant given the proximity of wildlife habitats to human settlements and agricultural land in biodiversity-rich regions of the world such as India (Ragothaman and Chirukandoth 2012). A thorough investigation of the immediate environment and circumstances of the mortality may raise suspicion of poisoning as a differential diagnosis. However, as is often the case in free-ranging wildlife mortalities, carcasses may be too decomposed at the time of detection for a necropsy to pinpoint the cause of death. Toxicological analyses can help to rule out poisoning or identify other causes for concern for wildlife, environmental and human health. Wildlife could even act as sentinels of environmental contamination, exemplified in this report by the detection of compounds such as endosulfan and imidacloprid. Wildlife researchers must make full use of the toxicological testing services provided free of charge by government laboratories like the RCEL in Kerala, and possibly other Indian states, to investigate the occurrence and impacts of poisoning.

Finally, the study highlights how pesticides continue to be easily available and inadequately regulated in India. Their use in public responses to human-wildlife conflict in India has been reported previously (Madhusudan 2003; Cheeran 2007; Venkataramanan et al. 2008). Since being banned in India in 2011 (Dhillon 2017), endosulfan has continued to be available for agricultural use, and human exposures have also been reported (Menezes et al. 2017), highlighting the lax implementation of existing regulations. The frequent involvement of such toxic compounds in animal and human deaths in India points to the need for strengthening legislation that regulates their availability and use without affecting their application in agricultural and allied sectors (Ragothaman and Chirukandoth 2012). Doing so will have wide-ranging benefits, extending beyond wildlife health and conservation to human and environmental health.

## Electronic supplementary material

Supplementary Table 1 (PDF 15.8 kb)
