# Supplementary material for: A note on wildlife poisoning cases from Kerala, South India
Source: Eur J Wildl Res. 2018 Sep 22;64(5):58. doi: 10.1007/s10344-018-1218-6 (PMC6390940; doi:10.1007/s10344-018-1218-6)
Supplement: Supplementary file 1 — (PDF 15.8 kb) [file 10344_2018_1218_MOESM1_ESM.docx]

**A note on wildlife poisoning cases from Kerala, South India**

European Journal of Wildlife Research

Sreejith Radhakrishnan^1,2*^

^1^Former Assistant Forest Veterinary Officer, Periyar Tiger Reserve, Department of Forests and Wildlife, Kerala, India

^2^Department of Infectious Disease Epidemiology, Imperial College London, St. Mary’s campus, Norfolk place, London W2 1PG, United Kingdom

[*sr1614@imperial.ac.uk](mailto:*sr1614@imperial.ac.uk)

**Table S1** Wildlife mortality events in Kerala, South India, recorded between January 2011 and March 2013 at the office of the Assistant Forest Veterinary Officer, Periyar Tiger Reserve, where poisoning was considered in the differential diagnoses, with details of the time period when the mortality event occurred, species and numbers affected, results of the toxicological analyses and the cause of death.

| **Sl. No.** | **Period** | **Species affected (Numbers affected)** | **Results of toxicological analyses** | **Cause of death** |
| --- | --- | --- | --- | --- |
| **1** | January 2011 | Tiger  (*Panthera tigris*) (1) | Negative | Natural causes |
| **2** | April 2011 | Elephant  (*Elephas maximus*) (1) | Negative | Undetermined, carcass decomposed |
| **3** | August 2011 | Elephant (4) | Negative | Lightning strike |
| **4** | October 2011 | Bonnet macaque  (*Macaca radiata*) (1) | No samples analysed | Carbofuran poisoning |
| **5** | November 2011 | Elephant (1) | Positive for imidacloprid | Imidacloprid poisoning |
| **6** | December 2011 | Leopard  (*Panthera pardus*) (1) | Negative | Undetermined – carcass decomposed |
| **7** | January 2012 | Dog (*Canis familiaris*) (1), Buffalo (*Bubalus bubalis*) (1), Wild boar (*Sus scrofa*) (3) | Negative | Unidentified haemorrhagic pathology |
| **8** | March 2012 | Elephant (1) | Negative | Undetermined, carcass decomposed |
| **9** | April 2012 | Elephant (1) | Negative | Unidentified pathology |
| **10** | August 2012 | Gaur  (*Bos gaurus*) (1) | Positive for endosulfan | Undetermined, carcass decomposed |
| **11** | September 2012 | Wild Boar (4) | Positive for warfarin | Warfarin poisoning |
